# Metagenome Sequencing Reveals *Rhodococcus* Dominance in Farpuk Cave, Mizoram, India, an Eastern Himalayan Biodiversity Hot Spot Region

**DOI:** 10.1128/genomeA.00610-15

**Published:** 2015-06-11

**Authors:** Surajit De Mandal, Zothan Sanga, Nachimuthu Senthil Kumar

**Affiliations:** Department of Biotechnology, Mizoram University, Aizawl, Mizoram, India

## Abstract

The present study employed 16S rRNA amplicon sequencing to survey the prokaryotic microbiota on Farpuk Cave, revealing a diverse bacterial community with 4,021 operational taxonomical units (OTUs), mainly dominated by the genus *Rhodococcus*. Moreover, 18.17% of the OTUs were unclassified at the phylum level, suggesting the existence of novel bacterial species.

## GENOME ANNOUNCEMENT

Mizoram, India, falling under the biodiversity hot spot regions of Eastern Himalayas, is not well known for its geologically unique cave habitats (1). Farpuk Cave provides unique opportunities to understand the community structure of the large reservoirs of unknown microbial life. In the present study, we performed 16S rRNA amplicon metagenomic sequencing of sediment samples collected from Farpuk Cave located in Champhai, Mizoram, in northeast India (23.10N 92.53E). Sediment samples were collected in sterilized containers from 10 sites of Farpuk Cave, and the genomic DNA was extracted using the FastDNA spin kit for soils (MP Biomedicals, Solon, OH, USA) and finally mixed to prepare a composite sample. The V3 hypervariable region of the 16S rRNA gene was amplified using the F341/R518 primer combination, and amplicon metagenomic sequencing was performed using the Illumina MiSeq platform followed by the analysis and annotation of output data using the QIIME data analysis package (2, 3). The sequencing yield was 381.05 Mb of data consisting of 1,261,787 reads and a G+C content of 59.03%. The average base quality (Phred score) was 36.79, and the individual sequence length was 150 bp. After removing singletons (abundances <2) and chimeras, 875,614 preprocessed consensus V3 reads were grouped into 4,021 operational taxonomical units (OTUs) at a similarity threshold of 0.97 using UCHIME and UCLUST (4, 5). The OTU representative sequence was aligned using the PyNAST tool (6), and the reference sequence of each OTU was classified using the Ribosomal Database Project (RDP) classifier and Greengenes OTU database (7, 8).

Among the 11 phyla detected in the cave metagenome, *Actinobacteria* (81.43%) was the most abundant phylum of bacteria. Other sequences were classified as follows: *Firmicutes* (10.41%), *Proteobacteria* (2.83%), *Acidobacteria* (2.39%), *Gemmatimonadetes* (0.30%), and *Bacteroidetes* (0.14%). These detected phyla were common inhabitants of the cave microbial community and found in other subsurface environments (9, 10). About 79.48% of the identified genera fell under *Rhodococcus*, an aerobic, nonsporulating, nonmotile Gram-positive bacterium that can catabolize a wide range of compounds. They are also known to produce bioactive steroids, acrylamide, and acrylic acid and are involved in fossil fuel biodesulfurization (11, 12). *Rhodococcus fascians*, *Propionibacterium acnes*, *Glaciecola polaris*, *Mycobacterium celatum*, *Virgisporangium ochraceum*, *Actinomadura vinacea*, and *Bacillus foraminis* were the main bacterial species in the cave sediments. However, a large number of reads did not classify at the phylum level, suggesting the existence of novel bacteria in Farpuk Cave. Further studies with whole-metagenome sequencing will resolve the industrially important novel genes and metabolic pathways.

### Nucleotide sequence accession number.

The sequences obtained in this project have been deposited in the NCBI Short Read Archive under the accession no. SRP057997.
